# Amino acid infusion blocks renal tubular uptake of an indium-labelled somatostatin analogue.

**DOI:** 10.1038/bjc.1993.266

**Published:** 1993-06

**Authors:** P. J. Hammond, A. F. Wade, M. E. Gwilliam, A. M. Peters, M. J. Myers, S. G. Gilbey, S. R. Bloom, J. Calam

**Affiliations:** Department of Medicine, Royal Postgraduate Medical School, Hammersmith Hospital, London, UK.

## Abstract

**Images:**


 © Macmillan Press Ltd., 1993

### Amino acid infusion blocks renal tubular uptake of an indium-labelled somatostatin analogue

P.J. Hammond, A.F. Wade, M.E. Gwilliam, A.M. Peters, M.J. Myers, S.G. Gilbey, S.R. Bloom & J. Calam

*Department of Medicine, Royal Postgraduate Medical School, Hammersmith Hospital, London W12 0NN, UK.*

**Summary** The Indium-labelled somatostatin analogue pentetreotide has been successfully developed for imaging of somatostatin receptor positive tumours. However there is significant renal tubular uptake of the radiolabelled peptide, which can obscure upper abdominal tumours and would preclude its use for targeted radiotherapy. The aim of this study was to determine whether amino acid infusion, which has been shown to block renal tubular peptide reabsorption, diminishes renal parenchymal uptake of this radiolabelled analogue.

Eight patients being scanned with the $^{111}$In-labelled somatostatin analogue, pentetreotide, for localisation of gastroenteropancreatic tumours received an infusion of synthetic amino acids. The ratio of isotope uptake in kidney to that in spleen was assessed, and compared to the ratio for matched control patients, to determine if amino acid infusion reduced renal parenchymal uptake of the radiopharmaceutical. The amount of isotope in the urine was determined to ensure that any effect of the amino acid infusion was unrelated to changes in clearance.

Infusion of amino acids significantly reduced renal parenchymal uptake of isotope at 4 h. There was a non-significant increase in urinary clearance of isotope over the 4 h, consistent with reduced reuptake and a lack of effect on glomerular filtration rate.

This technique, by preventing renal damage, may allow the use of this somatostatin analogue for local radiotherapy, and could be of wider value in blocking tubular re-uptake of potentially nephrotoxic agents, such as radiolabelled Fab fragments.

The $^{111}$In-labelled somatostatin analogue, pentetreotide, has recently been developed for the imaging of somatostatin receptor positive lesions, particularly gastroenteropancreatic tumours. In healthy volunteers up to 90% of this radiolabelled peptide is cleared by the kidney, 50% within the first 4.5 h. However by 4 h about 7% of the injected dose was taken up into the renal parenchyma, and about 5% remained at 48 h with a median absorbed dose of 0.45 mGy/MBq (range 0.19–0.8), and this uptake reduces the sensitivity of detection for small tumours located in the upper abdomen (Krenning *et al.*, 1992). Thus reduced renal uptake could enhance imaging sensitivity, and, furthermore, allow the use of analogues labelled with a $\beta$-emitting radionuclide, if these were to become available, for local radiotherapy without the risk of significant nephrotoxicity (Lamberts *et al.*, 1991).

The infusion of certain amino acids, particularly lysine and arginine, has been shown to block renal tubular peptide reabsorption (Morgenson & Sølling, 1977). The amino acids are thought to prevent binding between free positive amine or guanidine residues in the peptide and negatively charged sites on the surface of the renal tubule. Infusion of synthetic amino acids has been used therapeutically in acute renal failure (Abel *et al.*, 1973), although there are conflicting reports about its efficacy, with evidence that it can cause a deterioration in renal function in some patients (Zager, 1987). Infusion of a variety of amino acids in rats decreased glomerular filtration rate, the maximal effect being seen with arginine (58% decrease) and lysine (68% decrease). It was concluded that many amino acids are potentially nephrotoxic, and may sensitise the kidney to other forms of damage (Zager *et al.*, 1983). However in man infusion of an amino acid solution (Travasol) at a rate sufficient to cause a two-fold elevation in plasma amino acid concentrations (0.043 ml $kg^{-1} min^{-1}$) resulted in a 17% increase in glomerular filtration rate (GFR) and a 15% increase in renal plasma flow (RPF). Since amino acids stimulate release of a number of hormones with the potential for increasing RPF (e.g glucagon and growth hormone), somatostatin was infused at the same time as the amino acids, and this abolished the changes in RPF and GFR (Castellino *et al.*, 1987).

The aim of the present study was to determine whether amino acid infusion could reduce renal re-uptake of $^{111}$In-labelled pentetreotide, and to demonstrate that this was by reducing tubular re-uptake, rather than diminishing glomerular filtration rate. Such a technique of tubular uptake blockade could have widespread application in diminishing renal uptake of other potentially nephrotoxic agents: a similar phenomenon has been observed when magnesium is given with gentamicin to rats, magnesium competing with gentamicin for tubular binding sites and thus preventing gentamicin induced renal failure (Wong *et al.*, 1989).

### Materials and methods

Sixteen patients who were being imaged with $^{111}$In-labelled pentetreotide to localise a probable gastroenteropancreatic tumour were matched for age, sex, serum creatinine, tumour type and tumour bulk (Table I), and randomly allocated to the control or treatment groups. All had normal renal function.

$^{111}$In-labelled pentetreotide was kindly donated by Mallinckrodt Diagnostica (Petten, Holland). Injection of $^{111}$In-labelled pentetreotide was performed within 2 h of conjugating the indium and the peptide. The dose of $^{111}$In-labelled pentetreotide administered varied from 90 to 110 MBq. The effective whole body dose equivalent is 8 mSv/100 MBq, and so the maximum possible radiation dose was 8.8 mSv.

The amino acid preparation administered was Synthamin 14 without electrolytes, containing 4.93 g $l^{-1}$ lysine and 17.6 g $l^{-1}$ arginine and with a tonicity of 880 mosm $l^{-1}$, and this was obtained from Clintec (Slough, Berks., UK). Patients in the active group received this intravenously at a rate of 500 ml $h^{-1}$ over 4 h commencing at the time of $^{111}$In-labelled pentetreotide injection. All patients emptied their bladder immediately prior to commencing the infusion, and then voided at the end of the infusion to allow quantification of the amount of radioisotope cleared by the kidney during the infusion. Four hours after injection of the $^{111}$In-labelled pentetreotide all patients underwent plantar imaging of the upper abdomen using a gammacamera (Maxicamera IgE) with a medium energy collimator. At 4 h the radioisotope is

Correspondence: J. Calam.
Received 11 November 1992; and in revised form 26 January 1993.

predominantly peptide-bound in both plasma and urine (Krenning *et al.*, 1992). The ratio of kidney uptake to spleen uptake was assessed and quantified using a visual analogue scale from 1 to 5 (1 – spleen much greater than kidney, 3 – equal uptake, 5 – kidney much greater than spleen), in all cases by one person (AMP) blinded to the patient details. In four infused patients and their controls quantification was also performed by region of interest (ROI) analysis. ROI were drawn closely around the spleen and both kidneys. Radioactivity was expressed as (i) total counts per ROI, and (ii) as counts per pixel (unit area) for spleen and kidneys. The data was non-parametrically distributed and was analysed using a Mann Whitney U test. Results are expressed as mean ± standard error of the mean (s.e.m.), and $P<0.05$ is regarded as significant.

### Results

Using the visual analogue scale there was a highly significant 57% decrease in renal parenchymal uptake of isotope, when compared to spleen uptake, following amino acid infusion (Table II, Figure 1). There was close correlation between the kidney:spleen ratio as determined by this method and that calculated following ROI analysis ($r = 0.98$ for both methods of ROI analysis).

The significant decrease in renal parenchymal uptake in the infused patients was confirmed by quantification by counts by ROI analysis (Table III). Using total counts analysis there was a 66% reduction, and by counts per pixel analysis the reduction was 46%. There was no significant difference in splenic counts per pixel between the two groups (treated 86 ± 32 *vs* control 71 ± 45), even when adjusted for dose administered (treated 0.83 ± 0.28 *vs* control 0.69 ± 0.39 counts/MBq) and there was little correlation between the kidney:spleen ratio and the splenic uptake ($r = -0.45$).

The quantities of isotope in the urine collected during the 4 h post-injection were very similar in the two groups, with 63 ± 3% of the total dose cleared in the infused group and 61 ± 3% of the total dose cleared in the controls.

**Table I** Patients characteristics

| | *Control* | *Infused* |
|---|---|---|
| Age (years) | 55.9±13.7 (26–69) | 46.8±14.8 (27–69) |
| Sex | 2M, 6F | 1M, 7F |
| Creatinine (μmol l$^{-1}$) | 76.9±15.6 (61–111) | 72.5±14.1 (56–94) |
| Tumour type | 3 Carcinoid | 4 Carcinoid |
| | 3 Insulinoma | 1 Insulinoma |
| | 1 Gastrinoma | 2 Gastrinoma |
| | 1 Non-functioning | 1 Non-functioning |

**Table II** Comparison of renal uptake to splenic uptake in control and infused groups (mean ± s.e.m.) using visual analogue scale[a]; $^{b}P = 0.001$ *vs* control

| *Control* | *Infused* |
|---|---|
| 3.7±0.4 | 1.6±0.2[b] |

[a]Visual analogue scale: 1 Kidney<<Spleen, 2 Kidney<Spleen, 3 Kidney = Spleen, 4 Kidney>Spleen, 5 Kidney>>Spleen.

**Table III** Ratio of renal uptake to splenic uptake in control and infused groups (mean ± s.e.m [range]) by ROI analysis; $^{a}P = 0.02$ *vs* control

| | *Control* | *Infused* |
|---|---|---|
| Total counts | 2.03±0.76 | 0.69±0.10[a] |
| | (1.00–4.25) | (0.655–0.955) |
| Counts per pixel | 1.36±0.18 | 0.73±0.09[a] |
| | (0.985–1.8) | (0.485±0.94) |

### Discussion

This study demonstrates that amino acid infusion, which is thought to block renal tubular protein uptake, reduces the uptake of $^{111}$In-labelled pentetreotide by the kidney relative to the spleen. The similar uptake of isotope by the spleen in both groups indicates that this is the result of decreased renal

**Figure 1** Indium 111-labelled pentetreotide scans of **a**, control (visual analogue scale 5, kidney:spleen ratio 1.8) and **b**, infused patients (visual analogue scale 1, kidney:spleen ratio 0.48) showing uptake in kidney and spleen (arrowed).

re-uptake of $^{111}$In-labelled pentetreotide. The urinary clearance of isotope in the infused patients was marginally greater than that in the control group, indicating that the decreased renal uptake is due to blockade of the tubular uptake mechanism rather than the result of impaired glomerular filtration or attentuation of protein binding of the radiolabelled analogue. Furthermore the increase in urinary clearance of the isotope of about 2%, although not statistically significant since it represents such a small proportion of the total excreted dose (about 60% over the first 4 h), would be consistent with the degree of blockade of tubular re-uptake observed since the renal uptake is normally about 6% of the total dose and the reduction with amino acid infusion is about 50%.

Ninety percent of gastroenteropancreatic tumours carry a high density of somatostatin receptors. This radiolabelled analogue binds to these and thus effectively images these tumours (Krenning *et al.*, 1992; Lamberts *et al.*, 1991). In the future the analogue loaded with a β-emitting isotope could be used to give local radiotherapy. Both these uses could be enhanced by decreasing renal re-uptake: its sensitivity in detecting small tumours in the upper abdomen may be increased, and the reduced risk of damage to the renal parenchyma would make local radiotherapy more feasible.

The technique we have described could have more widespread application as a renal cytoprotectant. Cytoprotectants such as sodium thiosulphate, which reduces cisplatinum induced renal tubular necrosis, and the sulphhydryl-containing compounds N-acetylcysteine and 2-mercaptoethanesulfonate (Mesna), which block cyclophosphamide induced bladder toxicity, can significantly increase the value of compounds whose use is limited by toxicity towards normal tissue (Dorr, 1991). Infusion of amino acids could provide renal cytoprotection by blocking renal re-uptake of any toxic agents containing peptide fragments. One such application would be in the imaging and treatment of tumours using Fab fragments linked to radioisotopes or chemotherapeutic agents (Larson, 1990). Blocking Fab fragment re-uptake by concurrent infusion of amino acids would increase the target to non-target ratio for imaging purposes, since there is significant renal uptake: 6% of the total dose for $^{111}$In-OV-TL 3 $F(ab^1)_2$ which is used to image ovarian cancer (Buijs *et al.*, 1992). Therapeutic use of Fab fragments coupled to β-emitting radioisotopes or drugs would be less toxic if amino acid infusion could reduce nephrotoxicity from the coupled agents.

Furthermore inhibition of other tubular uptake mechanisms by appropriate receptor blockers has the potential to reduce nephrotoxicity from a variety of sources. These could include other radiolabelled compounds, such as DMSA, which could then be used to deliver local radiotherapy to mestatatic medullary carcinoma of the thyroid, chemotherapeutic agents, such as cisplatinum, and drug overdoses.

### References

ABEL, R.M., BECK, C.H. JR, ABBOTT, W.M., RYAN, J.A. JR, BARNETT, G.O. & FISCHER, J.E. (1973). Improved survival from acute renal failure after treatment with intravenous essential L-amino acids and glucose. Results of a prospective, double-blind study. *N. Engl. J. Med.*, **288,** 695–699.

BUIJS, W.C.A.M., MASSUGER, L.F.A.G., CLAESSENS, R.A.M.J., KENEMANS, P. & CORSTENS, F.H.M. (1992). Dosimetric evaluation of immunoscintigraphy using indium-111-labelled monoclonal antibody fragments in patients with ovarian cancer. *J. Nucl. Med.*, **33,** 1113–1120.

CASTELLINO, P., HUNT, W. & DEFRONZO, R.A. (1987). Regulation of renal haemodynamics by plasma amino acid and hormone concentration. *Kidney Int.*, **32,** S15–S20.

DORR, R.T. (1991). Chemoprotectants for cancer chemotherapy. *Seminars in Oncology*, **1** Suppl 2, 48–58.

KRENNING, E.P., BAKKER, W.H., KOOIJ, P.P., BREEMAN, W.A., OEI, H.Y., DE JONG, M., REUBI, J.C., VISSER, T.J., BRUNS, C., KWEKKEBOOM, D.J., REIJS, A.E.M., VAN HAGEN, P.M., KOPER, J.W. & LAMBERTS, S.W.J. (1992). Somatostatin receptor scintigraphy with indium-111-DTPA-D-Phe-1-octreotide in man: metabolism, dosimetry and comparison with iodine-123-Tyr-3-octreotide. *J. Nucl. Med.*, **33,** 652–658.

LAMBERTS, S.W.J., KRENNING, E.P. & REICH, J.-C. (1991). The role of somatostatin and its analogs in the diagnosis and treatment of tumors. *Endocrine Rev.*, **12,** 450–482.

LARSON, S.M. (1990). Clinical radioimmunodetection 1978–1988: overview and suggestions for standardization of clinical trials. *Cancer Res.*, **50,** 892S–898S.

MORGENSON, C.E. & SØLLING, K. (1977). Studies on renal tubular protein reabsorption: partial and near complete inhibition by certain amino acids. *Scand. J. Clin. Lab. Invest.*, **37,** 477–486.

WONG, N.L., MAGIL, A.B. & DIRKS, J.H. (1989). Effect of magnesium diet in gentamicin-induced acute renal failure in rats. *Nephron*, **51,** 84–88.

ZAGER, R.A., JOHANNES, G., TUTTLE, S.E. & SHARMA, H.M. (1983). Acute amino acid nephrotoxicity. *J. Lab. Clin. Med.*, **101,** 130–140.

ZAGER, R.A. (1987). Amino acid hyperalimentation in acute renal failure: a potential therapeutic paradox. *Kidney Int.*, **32,** S72–S75.


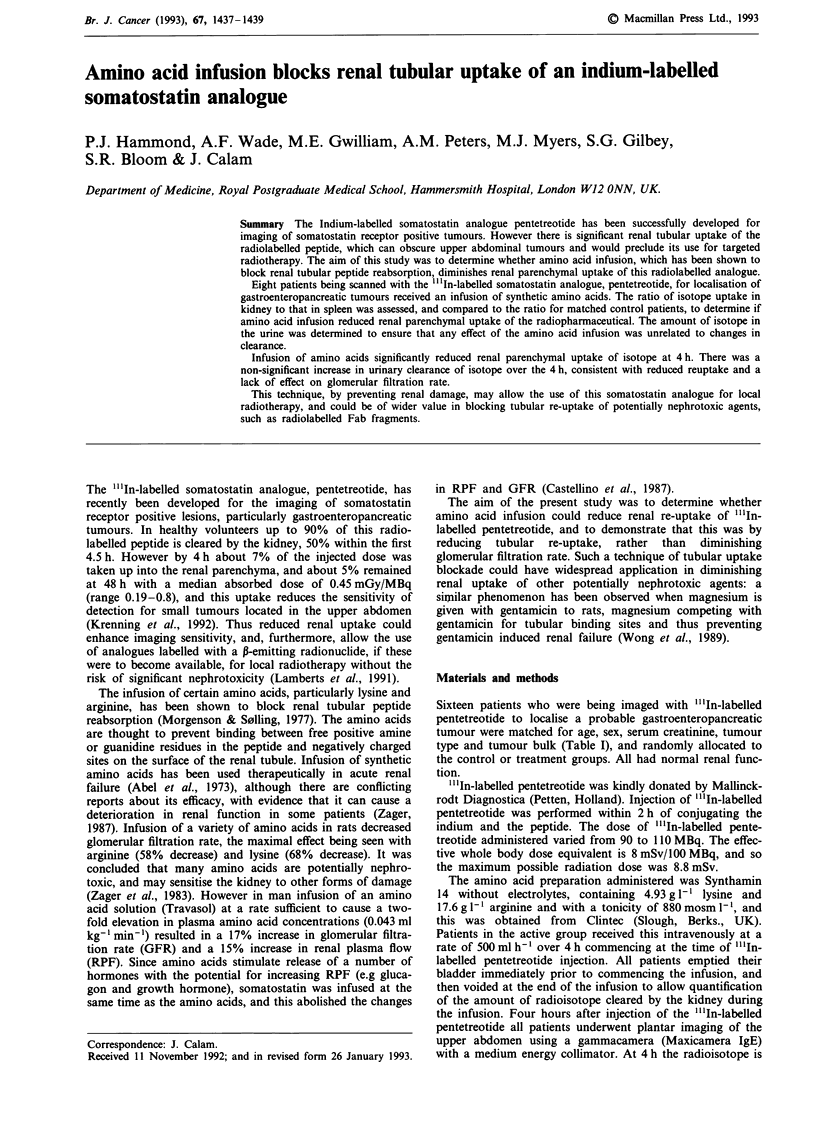

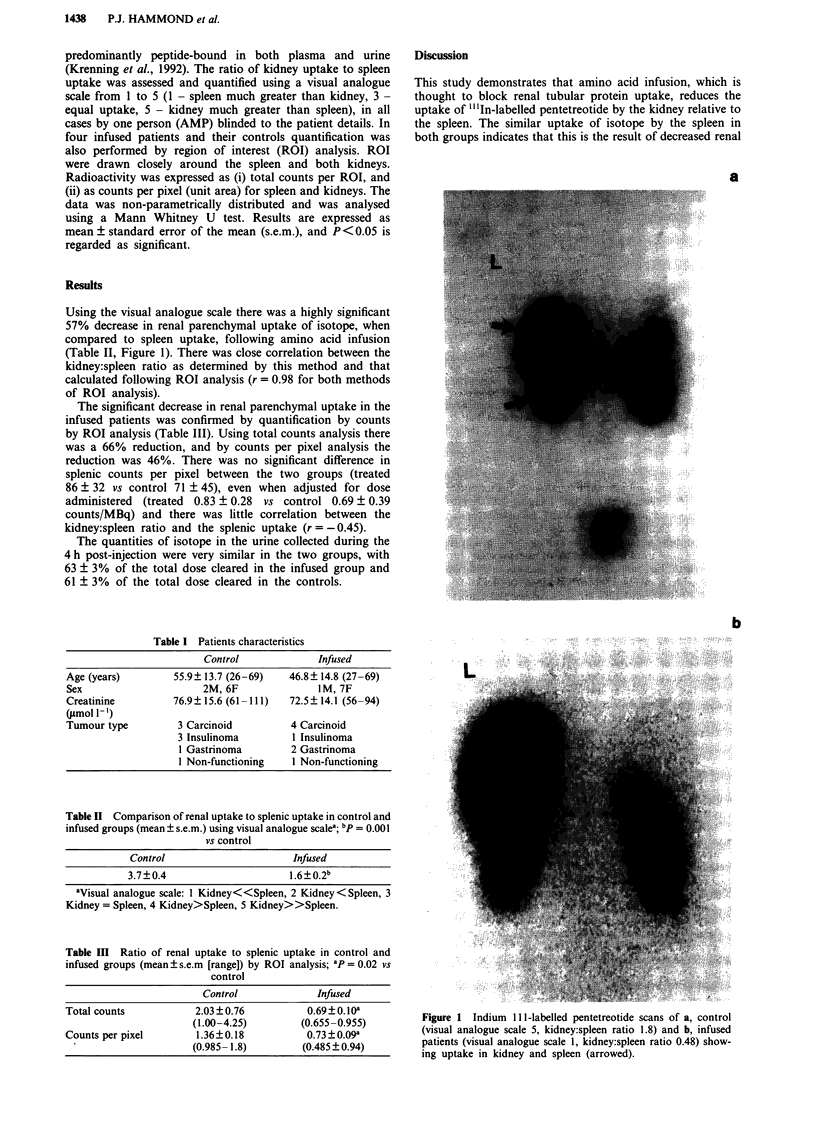

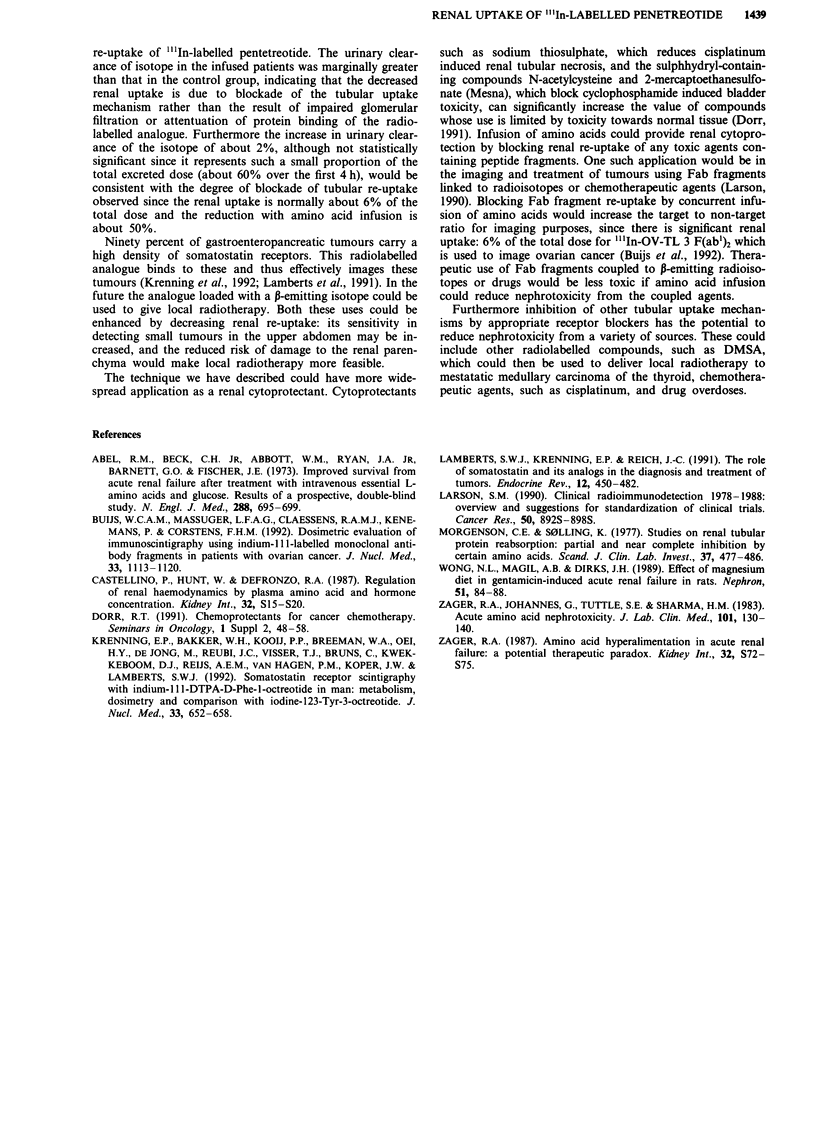


## References

[OCR_00290] Abel R. M., Beck C. H., Abbott W. M., Ryan J. A., Barnett G. O., Fischer J. E. (1973). Improved survival from acute renal failure after treatment with intravenous essential L-amino acids and glucose. Results of a prospective, double-blind study.. N Engl J Med.

[OCR_00299] Buijs W. C., Massuger L. F., Claessens R. A., Kenemans P., Corstens F. H. (1992). Dosimetric evaluation of immunoscintigraphy using indium-111-labeled monoclonal antibody fragments in patients with ovarian cancer.. J Nucl Med.

[OCR_00304] Castellino P., Hunt W., DeFronzo R. A. (1987). Regulation of renal hemodynamics by plasma amino acid and hormone concentrations.. Kidney Int Suppl.

[OCR_00309] Dorr R. T. (1991). Chemoprotectants for cancer chemotherapy.. Semin Oncol.

[OCR_00316] Krenning E. P., Bakker W. H., Kooij P. P., Breeman W. A., Oei H. Y., de Jong M., Reubi J. C., Visser T. J., Bruns C., Kwekkeboom D. J. (1992). Somatostatin receptor scintigraphy with indium-111-DTPA-D-Phe-1-octreotide in man: metabolism, dosimetry and comparison with iodine-123-Tyr-3-octreotide.. J Nucl Med.

[OCR_00322] Lamberts S. W., Krenning E. P., Reubi J. C. (1991). The role of somatostatin and its analogs in the diagnosis and treatment of tumors.. Endocr Rev.

[OCR_00327] Larson S. M. (1990). Clinical radioimmunodetection, 1978-1988: overview and suggestions for standardization of clinical trials.. Cancer Res.

[OCR_00332] Mogensen C. E., Sølling (1977). Studies on renal tubular protein reabsorption: partial and near complete inhibition by certain amino acids.. Scand J Clin Lab Invest.

[OCR_00336] Wong N. L., Magil A. B., Dirks J. H. (1989). Effect of magnesium diet in gentamicin-induced acute renal failure in rats.. Nephron.

[OCR_00346] Zager R. A. (1987). Amino acid hyperalimentation in acute renal failure: a potential therapeutic paradox.. Kidney Int Suppl.

[OCR_00341] Zager R. A., Johannes G., Tuttle S. E., Sharma H. M. (1983). Acute amino acid nephrotoxicity.. J Lab Clin Med.

